# Endothelial cell-specific molecule 1 drives cervical cancer progression

**DOI:** 10.1038/s41419-022-05501-5

**Published:** 2022-12-15

**Authors:** Jingjing Lu, Qin Liu, Lixia Zhu, Yuanyuan Liu, Xiaoren Zhu, Shiqing Peng, Minbin Chen, Ping Li

**Affiliations:** 1 Department of Radiotherapy and Oncology, Affiliated Kunshan Hospital of Jiangsu University, Kunshan, China; 2 Department of Gynaecology and Obstetrics, Affiliated Kunshan Hospital of Jiangsu University, Kunshan, China; 3 Clinical Research and Lab Center, Affiliated Kunshan Hospital of Jiangsu University, 215300 Kunshan, China

**Keywords:** Cancer therapy, Cell growth

## Abstract

The expression, biological functions and underlying molecular mechanisms of endothelial cell-specific molecule 1 (ESM1) in human cervical cancer remain unclear. Bioinformatics analysis revealed that *ESM1* expression was significantly elevated in human cervical cancer tissues, correlating with patients’ poor prognosis. Moreover, *ESM1* mRNA and protein upregulation was detected in local cervical cancer tissues and various cervical cancer cells. In established and primary cervical cancer cells, ESM1 shRNA or CRISPR/Cas9-induced ESM1 KO hindered cell proliferation, cell cycle progression, in vitro cell migration and invasion, and induced significant apoptosis. Whereas ESM1 overexpression by a lentiviral construct accelerated proliferation and migration of cervical cancer cells. Further bioinformatics studies and RNA sequencing data discovered that ESM1-assocaited differentially expressed genes (DEGs) were enriched in PI3K-Akt and epithelial-mesenchymal transition (EMT) cascades. Indeed, PI3K-Akt cascade and expression of EMT-promoting proteins were decreased after ESM1 silencing in cervical cancer cells, but increased following ESM1 overexpression. Further studies demonstrated that SYT13 (synaptotagmin 13) could be a primary target gene of ESM1. SYT13 silencing potently inhibited ESM1-overexpression-induced PI3K-Akt cascade activation and cervical cancer cell migration/invasion. In vivo, ESM1 knockout hindered SiHa cervical cancer xenograft growth in mice. In ESM1-knockout xenografts tissues, PI3K-Akt inhibition, EMT-promoting proteins downregulation and apoptosis activation were detected. In conclusion, overexpressed ESM1 is important for cervical cancer growth in vitro and in vivo, possibly by promoting PI3K-Akt activation and EMT progression. ESM1 represents as a promising diagnostic marker and potential therapeutic target of cervical cancer.

## Introduction

Cervical cancer is a common gynecological malignancy, the fourth-common cause of incidence and death of cancer among women [1–3]. The majority of cervical cancer patients (about 70%) with nonmetastatic disease could be cured with current therapies, including surgery, chemotherapy, and radiotherapy [4, 5]. Nearly 30% of cervical cancer patients with metastatic and recurrent cancers [2, 3, 6]. Due to the limited efficient strategies, the five-year overall survival rate for these advanced patients is less than 17% [3, 7]. Genomic mutation exists in over 70% of all cervical cancer patients [8], which attracts more interest in the development of molecularly-targeted agents [7, 8]. Therefore, the identification of novel and more efficient anti-cervical cancer therapeutics is desperately needed [3, 9–11].

Endothelial cell-specific molecule 1 (ESM1), also known asendocan, is an acysteine-rich proteoglycan. The *ESM1* gene is located on chromosome 5 q11.2 [12]. ESM1 was considered an endothelial cell-specific molecule expressed in the lung tissue [13]. Recent studies have reported that ESM1 is expressed in multiple tissues, including the kidney, liver, thyroid gland, skin, and gastrointestinal tract [14–16]. Moreover, aberrant ESM1 expression is associated with multiple diseases, including inflammation [17], vascular disorders [18], and cancer [19].ESM1 is upregulated in a broad spectrum of cancers, often correlated with a poor prognosis [19–22].

More recently, studies have found that ESM1 could participate in various physiological processes in cancer cells, including cell proliferation, angiogenesis, migration, and invasion, as well as therapy resistance. It therefore plays a crucial role in carcinogenesis and tumor progression [19, 23, 24]. Multiple proinflammatory cytokines and growth factors can enhance ESM1 expression [16]. In bladder cancer, ESM1 promoted cancer metastasis by positively regulating VEGF-A/VEGFR-2 axis [25]. Chronic intermittent hypoxia enhanced lung cancer stem cell progression and invasion by activating ESM1/HIF-1α cascade [26]. In addition, ESM1 was reported to promote cancer progression and invasion by regulating numerous pathways, including DLL4-Notch, NF-κB, PI3K-Akt-mTOR, and Wnt/β-catenin signalings in human adrenocortical carcinoma [22], colorectal cancer [27], breast cancer [28], glioma [29], and prostate cancer [19]. Interestingly, Chen *et al*. reported that ESM1 can suppress the progression and metastasis of prostate cancer cells by regulating TIMP-1/MMP-9 expression [30].

Epithelial-to-mesenchymal transition (EMT) plays a crucial role in cancer progression [31, 32], as it facilitates epithelial cells to acquire mesenchymal features, alters cell polarity, and weakens cell adhesion [33]. EMT promotes cancer cell invasion and drives tumor metastasis, recurrence, and participates in therapyresistance [31, 33]. In cervical cancer, EMT was often induced by oncogenes, hypoxia, and activation of certain transcription factors [26, 27, 32]. The induction, progression, and maintenance of EMT are vital in the progression and metastasis of cervical cancer [32]. EMT was regulatedby ESM1 in tumor invasion of colorectal cancer through the activation of the NF-κB pathway [27].In addition, non-small-cell lung cancer proliferation, stemness, and EMT were regulated by ESM1/HIF-1α pathway [26].The expression and potential functions of ESM1 in cervical cancer have not been studiedthus far.

## Results

### ESM1 is upregulated in human cervical cancer

**Fig. 1:**
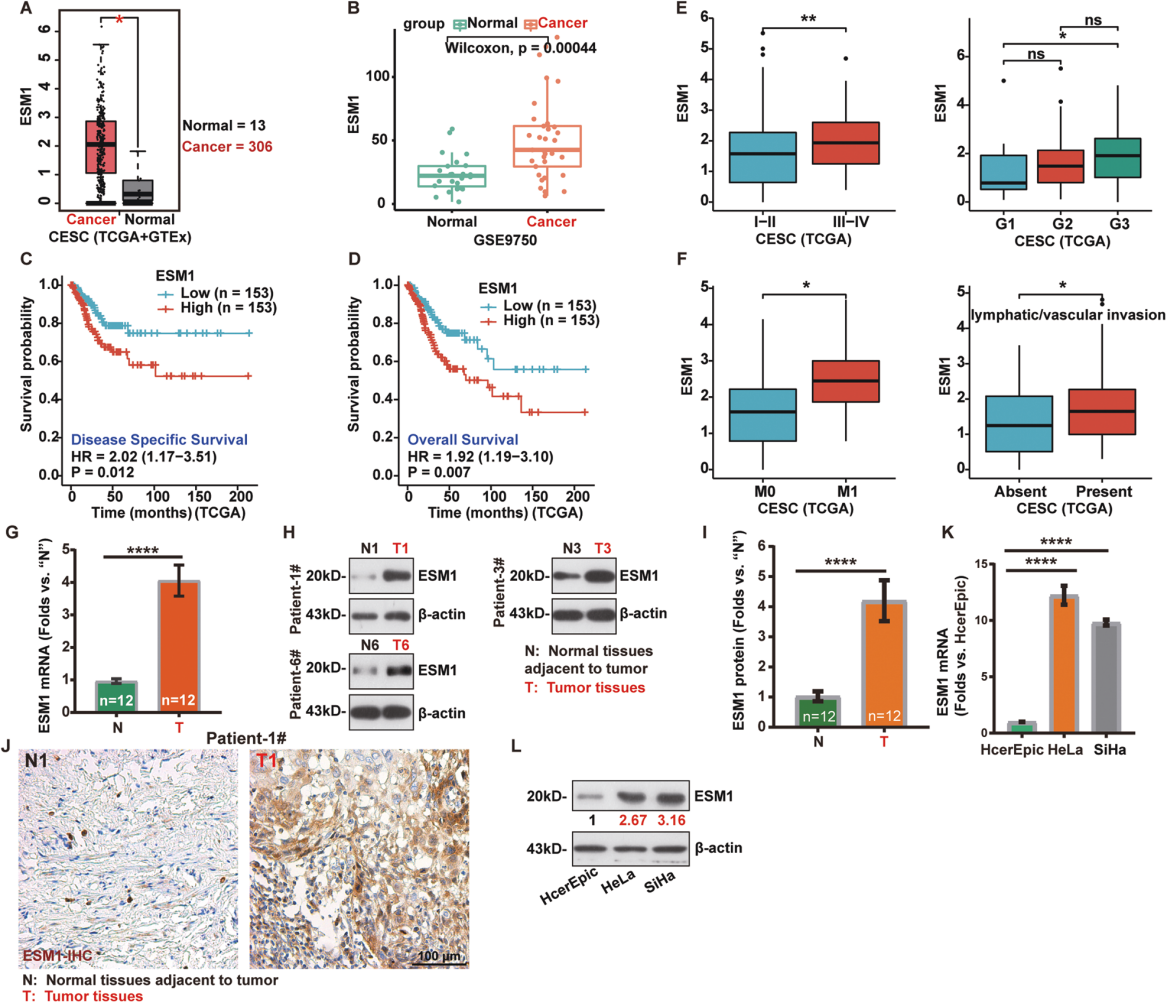
ESM1 is upregulated in human cervical cancer. The mRNA transcripts of ESM1 in cervical cancer (*n* = 306) tissues compared with normal tissues (*n* = 13) in TCGA database (**A**) and GEO database (**B**) were shown. Disease-specific survival (**C**) and overall survival (**D**) in cervical cancer patients with different expression levels of ESM1 were shown. Subgroup analysis, based on the different clinical characteristics of the cervical cancer patients in TCGA, was conducted (**E, F**). *ESM1* mRNA (**G**) and protein expression (**H** and **I**) in twelve (*n* = 12) pairs of cervical cancertumor tissues (“T”) and adjacent normal tissues (“N”) were tested by qRT-PCR and Western blotting assays. Immunohistochemical staining was used to test the expression of ESM1 in Patient-1#’s tissue (**J**). *ESM1* mRNA (**K**) and protein (**L**) expression in the listed cervical cancer cells and the cervical epithelial cell was tested by qRT-PCR and Western blotting assays. The data were presented as mean ± standard deviation (SD). *P < 0.05, **P < 0.01, ***P < 0.001, ****P < 0.0001. Scale bar = 100 μm (**J**).

The Cancer Genome Atlas (TCGA) database and the Genotype-Tissue Expression (GTEx) project were consulted to analyze *ESM1* transcripts in human cervical cancer tissues. A total of 319 tissues were collected, including 13 normal cervical tissue samples and 306 cervical cancer tissue samples. As shown, the number of *ESM1* mRNA transcripts in the cervical cancer tissues was significantly higher than that in the normal cervical tissues (Fig. 1A). We further explored ESM1 expression in cervical cancer by retrieving the Gene Expression Omnibus (GEO). *ESM1* mRNA was significantly upregulated in 33 cervical cancer tissues compared to 24 normal tissues in GSE9750 dataset (Fig. 1B). In addition, the association between *ESM1* expression and the prognosis of cervical cancer was analyzed based on TCGA database. Kaplan-Meier survival analysis showed that cervical cancer patients with low ESM1 expression had significantly longer disease-specific survival (DSS, Fig. 1C) and overall survival (Fig. 1D).

Subgroup analysis based on different clinical characteristics in TCGA database showed that high expression of ESM1 was positively correlated with the high clinicopathological stage (*P* < 0.01) and disease classification in cervical cancer patients (Fig. 1E). In addition, *ESM1* overexpression was closely associated with tumor metastasis (*P* < 0.05) and lymphatic vascular invasion (*P* <0.05) (Fig. 1F). Therefore, *ESM1* overexpression is correlated with poor overall survival and major clinicopathological parameters in cervical cancer.

To confirm the bioinformatics results, we tested ESM1 expression in cervical cancer tissues of local patients administrated at our hospital. The cervical cancer tissues (“T”) and paired adjacent normal tissues (“N”) were from a total of 12 different primary cervical cancer patients (*n* = 12). *ESM1* mRNA expression in cervical cancer tumor tissues was about four-fold higher than that in adjacent normal tissues (Fig. 1G). ESM1 protein was upregulated in cervical cancer tissues from three representative patients (Patient#1/#3/#6) (Fig. 1H). After combining all 12 sets of ESM1 blotting data, we found that ESM1 protein expression is significantly upregulated in the cervical cancer tissues (*P* < 0.0001 vs. “N” tissues, Fig. 1I). The representative immunohistochemistry (IHC) assay results further verified ESM1 protein upregulation in cervical cancer tissues of Patient#1 (Fig. 1J). Therefore ESM1 is upregulated in local cervical cancer tissues.

Moreover, ESM1 expression in cervical cancer cells was tested. *ESM1* mRNA expression was significantly higher in established HeLa and SiHa cell lines than that in normal cervical epithelial cells (HcerEpic)(Fig. 1K). Furthermore, ESM1 protein was upregulated as well in established cervical cancer cells when compared to that in normal cervical epithelial cells (Fig. 1L). Collectively, these results confirmed that ESM1 is upregulated in cervical cancer cells.

### ESM1 knockdown inhibits cervical cancer cell progression in vitro

**Fig. 2:**
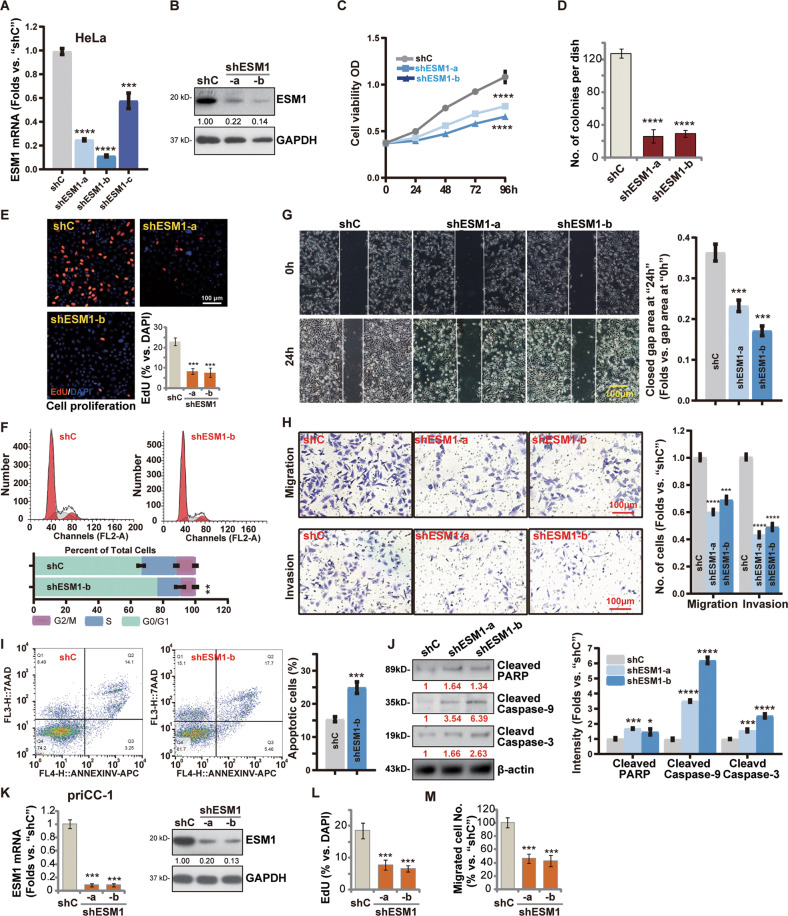
ESM1 knockdown inhibits cervical cancer cells progression. Established cervical cancer cell line (HeLa) (**A**–**J**) and primary cervical cancer cells (“priCC-1”) (**K**–**M**) that expressed the ESM1 shRNA (shESM1-a/shESM1-b/shESM1-c) or the scramble control shRNA (“shC”) were established and cultured. The effects of ESM1 knockdown were confirmed by qRT-PCR (**A** and **K**) and Western blotting (**B** and **K**), GAPDH was used as an internal control. Cell viability was tested by CCK8 assay. The OD value was detected at 0 h, 24 h, 48 h, 72 h, and 96 h (**C**). Cell growth was tested by colony formation assay (**D**). EdU staining was used for cell proliferation (**E** and **L**). Three random views (*n* = 3) of total 1, 000 cell nuclei per each condition were used to calculate the average EdU ratio (% vs. DAPI), and same for all EdU studies. Cell cycle progression was detected by flow cytometry (**F**). Wound-healing assay was used to examine the migration effect of ESM1 (**G**). The wound space was photographed at 0 h, and 24 h (**G**). Cell migration and invasion were tested by Transwell and Matrigel Transwell assays (**H** and **M**). Three random microscopy views were included to calculate the average number of migrated or invaded cells in each condition (same for all “Transwell” assays). Apoptosis was detected by flow cytometry (**I**). Caspase-PARP activationwas tested by Western blotting assay (**J**). The data were presented as mean ± standard deviation (SD, *n* = 3). *P < 0.05, **P < 0.01, ***P < 0.001, ****P < 0.0001 vs. “shC” cells. The experiments were repeated three times with similar results obtained. Scale bar = 100 μm.

To investigate the function of ESM1 in cervical cancer cells, the shRNA strategy was employed to knockdown ESM1. HeLa cells were transfected with each of the three different lentiviral shRNAs targeting non-overlapping sequences of ESM1. The three were named shESM1-a, shESM1-b, and shESM1-c. Stable cells were established by selection with puromycin. In shESM1-a /shESM1-b-expressing stable cervical cancer cells, *ESM1* mRNA expression was decreased significantly (Fig. 2A). The ESM1 protein expression was significantly downregulated as well by shESM1-a and shESM1-b (Fig. 2B). CCK-8 assay results showed that the viability of cervical cancer cells was significantly decreased by silencing of ESM1 (Fig. 2C). The colony formation assay further demonstrated the growth of shESM1-expressing stable cervical cancer cells was largely inhibited and the number of colonies was reduced (Fig. 2D). In addition, cervical cancer cell proliferation was inhibited by the applied ESM1 shRNAs, leading to the significantly decreased percentage of EdU-positive nuclei (Fig. 2E).

The PI-FACS assays demonstrated that ESM1 knockdown by shESM1-b induced reduction of S-phase cells but the increase of G1-phase cells (Fig. 2F), implying that ESM1 silencing induced G1-S arrest. The wound-healing assay (Fig. 2G) as well as “Transwell” and “Matrigel Transwell” assays (Fig. 2H) showed that ESM1 shRNA potently inhibited cervical cancer in *vitro* cell migration and invasion.

Next, we tested the potential effect of ESM1 silencing on cell apoptosis. FACS assay results revealed that ESM1 silencing led to significantly increased cervical cancer cells with Annexin V-7AAD positive staining (Fig. 2I). Furthermore, increased cleavages of PARP, caspase-9, and caspase-3 were detected in the shESM1-expressing stable HeLa cells (Fig. 2J).

The potential effect of ESM1 silencing on the primary human cervical cancer cells (“priCC-1”) was studied. shESM1-a and shESM1-b led to robust *ESM1* mRNA and protein (Fig. 2K) downregulation in priCC-1 primary cells. Functional studies revealed that ESM1 silencing by the applied shRNAs robustly inhibited priCC-1 cell proliferation (nuclear EdU incorporation reduction, Fig. 2L) and in vitro cell migration (see quantified results in Fig. 2M).

### ESM1 knockout exerts potent anti-cervical cancer cell activity

To further validate the pivotal role of ESM1 in cervical cancer cells, the CRISPR/Cas9 strategy was employed to genetically knockout ESM1. A lentiviral CRISPR/Cas9-ESM1-KO construct was designed and transduced to Cas9-expressing cervical cancer cells. We established stable CRISPR/Cas9-ESM1-KO cells through flow cytometry sorting and subsequent *ESM1* KO screening. Compared to the control cells with the CRISPR/Cas9 empty vector (“Cas9-C”) *ESM1* mRNA expression was significantly decreased in the koESM1-cervical cancer cells (“-a/-b/-c” stands for three selections, Fig. S2A). ESM1 KO decreased the viability (Fig. S2B) and inhibited colony formation (Fig. S2C) in HeLa and SiHa cells. CRISPR/Cas9-mediated ESM1 KO inhibited cervical cancer cell proliferation (EdU-nuclei ratio decreasing, Fig. S2D) and cell cycle progression (causing G1-S arrest, Fig. S2E). In addition, ESM1 KO dramatically suppressed cervical cancer cell in vitro migration and invasion (Fig. S2F and G). Further functional studies revealed that ESM1 KO increased Annexin V staining in cervical cancer cells, supporting apoptosis activation (Fig. S2H).

The potential effect of ESM1 KO on priCC-1 primary cells was explored. The CRISPR/Cas9-ESM1-KO construct was stably transduced to priCC-1 primary cells, resulting in the complete depletion of *ESM1* mRNA (Fig. S2I) and protein (Fig. S2I). In consistent with the results in established cell lines, we found that ESM1 KO robustly suppressed priCC-1 cell proliferation (Fig. S2J) and in *vitro* cell migration (Fig. S2K).

### Ectopic overexpression of ESM1 exerts significant promotion on cervical cancer cell progression

**Fig. 3:**
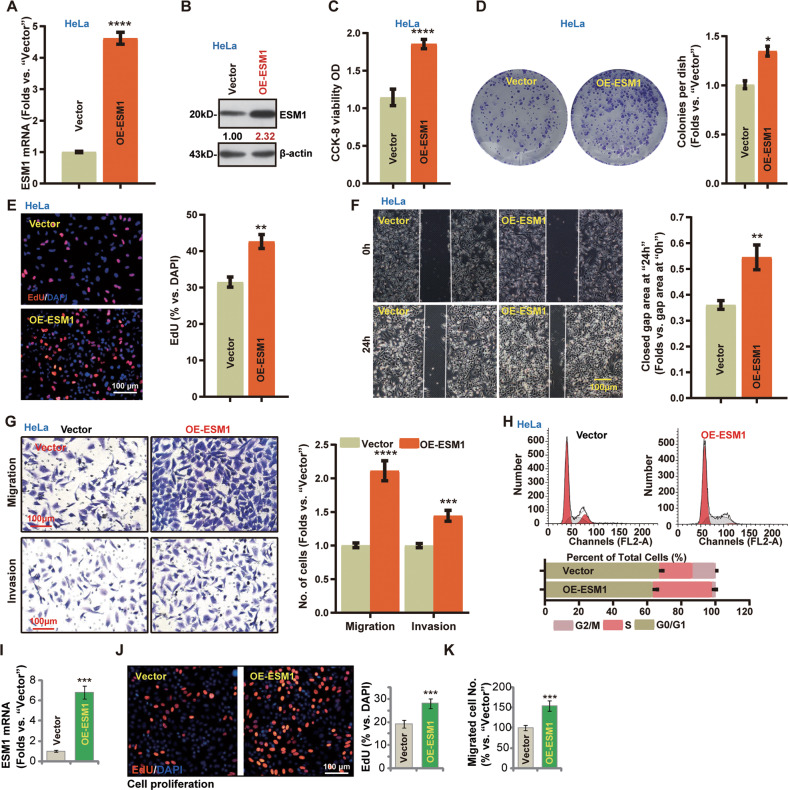
Ectopic overexpression of ESM1 exerts significant promotion on cervical cancer cell progression. Lentiviral constructs encoding the full-length ESM1 cDNA (“OE-ESM1”) and the empty vector (“Vector”) were established and were stably transduced to established cervical cancer cell lines (HeLa) (**A**–**H**) and primary cervical cancer cells (“priCC-1”) (**I**–**K**). ESM1 overexpression was confirmed by qRT-PCR (**A** and **I**) and Western blotting (**B**) assays. Cell viability (**C**), colony formation (**D**), proliferation (**E** and **J**), in vitro cell migration and invasion (**F**, **G** and **K**), as well as cell cycle progression (**H**), were tested by the listed assays, with results quantified. The data were presented as mean ± standard deviation (SD, *n* = 3). *P < 0.05, **P < 0.01, ***P < 0.001, ****P < 0,0001 vs. “Vector” cells. The experiments were repeated three times with similar results obtained. Scale bar = 100 μm.

Next, the lentivirus with the ESM1 cDNA-expressing construct was transduced to HeLa cells. Puromycin was added to select stable cells: namely OE-ESM1 cells. As compared to the control cells with the empty vector (“Vector”) *ESM1* mRNA and protein levels were remarkably upregulated in the OE-ESM1 cells (Fig. 3A and B). ESM1 overexpression in HeLa cells enhanced the CCK-8 viability OD (Fig. 3C) and promoted colony formation (Fig. 3D). Moreover, ESM1 overexpression enhanced HeLa cell proliferation (Fig. 3E), in vitro cell migration and invasion (Fig. 3F and G). In addition, ESM1 overexpression increased the percentage of cells in S-phase (Fig. 3H). The ESM1 cDNA-expressing lentiviral construct was transduced to priCC-1 primary cells and caused *ESM1* mRNA upregulation (Fig. 3I). Ectopic overexpression of ESM1 accelerated priCC-1 cell proliferation (Fig. 3J) and in vitro cell migration (Fig. 3K). Together, ectopic ESM1 overexpression promoted cervical cancer cell progression in vitro.

### ESM1-driven PI3K-Akt activation and EMT progression are possibly due to promoting SYT13 expression in cervical cancer cells

**Fig. 4:**
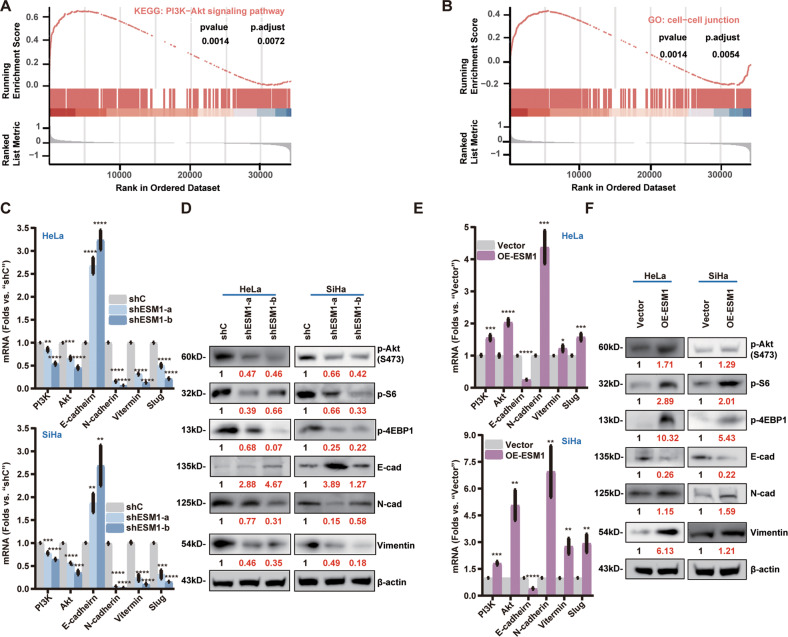
ESM1 activates the PI3K signaling pathway and facilitate EMT in cervical cancer cells. Gene Set Enrichment Analysis of Gene Ontology (**A**) and pathway enrichment analyses (**B**) of differential expression genes (DEGs) between high-ESM1 and low-ESM1 expression cervical cancer tissues from TCGA database. *PI3K*, *Akt*, *E-cadherin*, *N-cadherin*, *Slug*, and *Vimentin* mRNAs were detected by qRT-PCR (**C** and **E**) in the described cervical cancer cells with the applied genetic modifications. Expression of the listed proteins was tested by Western blotting assays (**D** and **F**). The data were presented as mean ± standard deviation (SD, *n* = 3). *P < 0.05, **P < 0.01, ***P < 0.001, ****P < 0,0001 vs. “shC”/“Vector” cells. The experiments were repeated three times with similar results obtained.

To explore the possible molecular mechanisms underlying ESM1-driven cervical cancer cell progression. Gene Set Enrichment Analysis (GSEA) was employed to analyze the differentially expressed genes (DEGs), by comparing ESM1 high-expression cervical cancer tissues with the ESM1 low-expression cervical cancer tissues retrieved from TCGA database. As shown, DEGs were enriched in PI3K-Akt cascade (Fig. 4A) and cell-cell junctions (Fig. 4B). PI3K-Akt-mTOR axis activation can promote proliferation and growth of epithelial cancer cells [34] and can enhance cell invasion and metastatic progression [35]. EMT was regulated by cell-cell junctions [36]. In the procession of EMT cell adhesion was decreased, facilitating cell migration and invasion [36].

As shown, Akt, S6, and 4EBP1 phosphorylation as well as mTOR and PI3K expression were significantly downregulated in shESM1-expressing cervical cancer cells (Fig. 4C and D). While ectopic overexpression of ESM1 dramatically increased Akt-mTOR activation in cervical cancer cells (Fig. 4E andF). EMT markers were examined as well. shRNA-mediated silencing of ESM1 downregulated the expression of Vimentin, N-Cadherin and Slug, while upregulating E-Cadherin in cervical cancer cells (Fig. 4C and D). Contrarily, ectopic ESM1 overexpression exerted the opposite activity on the expression of these EMT marker genes (Fig. 4E and F). Thus, ESM1 overexpression is important for PI3K-Akt- mTOR activation and EMT in cervical cancer cells.

**Fig. 5:**
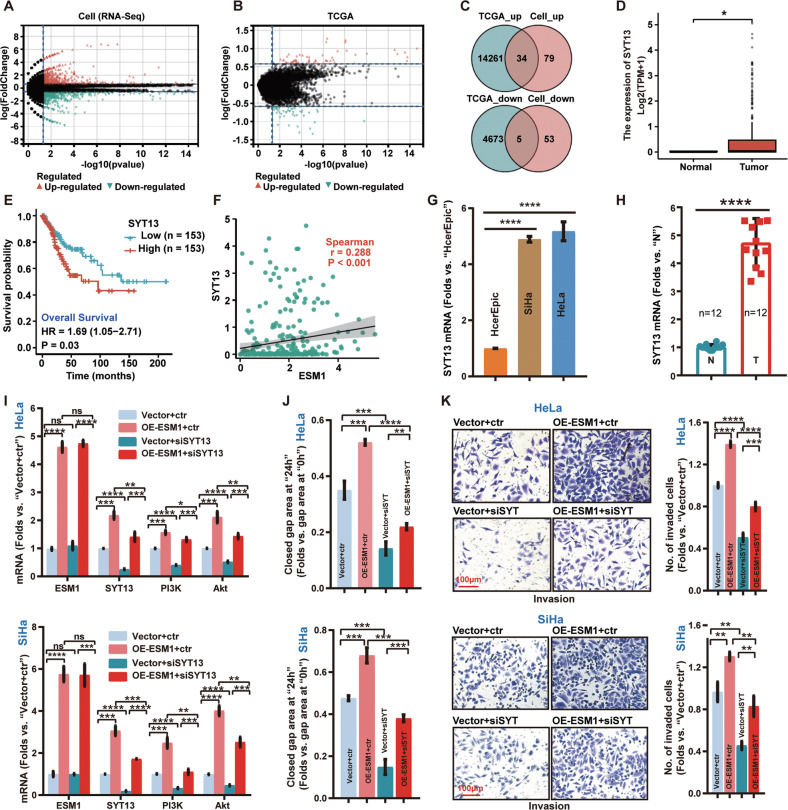
ESM1 activates the PI3K signaling pathway via up-regulating SYT13 expression in cervical cancer cells. RNA sequencing revealed differential expression genes (DEGs) between in ESM1-overexpressed (“OE-ESM1”) and vector control (“Vector”) SiHa cells (**A**); DEGs based on ESM1 expression in TCGA cervical cancer tissues (**B**). Venn diagram of common upregulated and downregulated genes in TCGA database and modified cells (**C**). *SYT13* mRNA expression in cervical cancer tissues (*n* = 306) and in normal tissues (*n* = 13) from TCGA database (**D**). Overall survival in cervical cancer patients with different ESM1 expressions (**E**). Correlation between *ESM1* and *SYT13* expression in TCGA cervical cancer tissues (**F**). *SYT13* mRNA expression in cervical cancer cells and cervical epithelial cells was tested (**G**). *SYT13* mRNA expression in cervical cancer (“T”, *n* = 12) and adjacent normal cervical tissues (“N”, n = 12) was tested (**H**). ESM1-overexpressed (“OE-ESM1”) and vector control (“Vector”) cervical cancer cells were further transfected with SYT13 siRNA (“siSYT13”) or scramble control siRNA (“ctr”) for 48 h, expression of listed mRNAs was shown (**I**); In vitro cell migration and invasion were tested (**J** and **K**), and results were quantified. The data were presented as mean ± standard deviation (SD, *n* = 3). *P < 0.05, **P < 0.01, ***P < 0.001, ****P < 0.0001. The experiments were repeated three times with similar results obtained. Scale bar = 100 μm.

Next, the bioinformatics analysis was performed in OE-ESM1 and control SiHa cells (see Fig. 3). DEGs between the high ESM1 expression cells (OE-ESM1) and vector control cells (Vector) were analyzed by R-software. As shown, mRNA transcripts of 113 genes were significantly upregulated in OE-ESM1 cells, and 58 mRNA transcripts were downregulated (Fig. 5A). In addition, we also explored the DEGs based on ESM1 expression in TCGA cervical cancer database. It was revealed that mRNA transcripts of 14295 genes were upregulated, while 4678 genes were downregulated (Fig. 5B). Subsequently, the common DEGs were further analyzed, and 34 genes were upregulated and five genes were downregulated (Fig. 5C).

Among the upregulated genes, *SYT13* (synaptotagmin 13) expression was significantly higher in cervical cancer tissues (*n* = 306) than that in normal cervical tissues (*n* = 13) (Fig.5D). Moreover, the Kaplan–Meier survival curves demonstrated that SYT13 overexpression was correlated with poor patients’ overall survival (Fig. 5E). Next, the spearman correlation between ESM1 and SYT13 was performed, and a positive correlation was detected *(r* = 0.288, *P* < 0.001, Fig. 5F). In addition, *SYT13* mRNA expression was much higher in cervical cancer cells than that in cervical epithelial cells (Fig. 5G). Moreover, *SYT13* mRNA expression was increased in cervical cancer tissues compared to that in the adjacent noncancerous tissues (Fig. 5H).

Significantly, SYT13 was upregulated in ESM1-overexpressed SiHa and HeLa cervical cancer cells (OE-ESM1, Fig. 5I). Remarkably, siRNA-mediated silencing of SYT13 reduced *SYT13* mRNA expression, and strongly inhibited PI3K-Akt cascade, leaving ESM1 expression unaffected (Fig. 5I). SYT13 siRNA suppressed migration and invasion of both vector control and ESM1-overexpressed cervical cancer cells (Fig. 5J and K). Together, ESM1 overexpression-induced PI3K-Akt activation as well as cervical cancer cell invasion/ migration might be due to increasing SYT13 expression.

### ESM1 knockdown inhibits cervical cancer cell growth in vivo

**Fig. 6:**
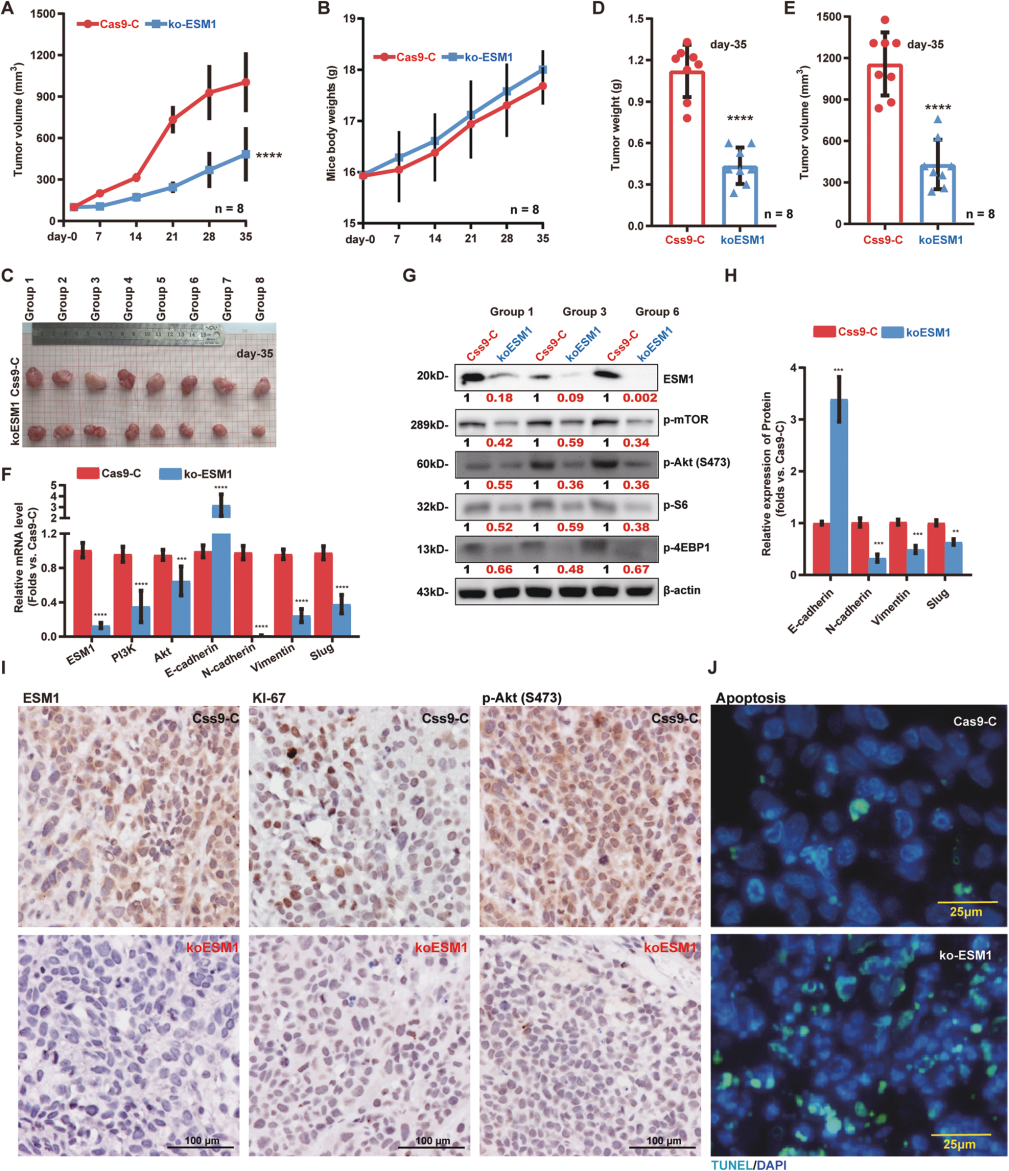
ESM1 knockout inhibits cervical cancer xenograft growth in mice. SiHa xenograft-bearing female BALB/c nude mice were established by subcutaneous injection of SiHa cells with the lentiviral CRISPR/Cas9-ESM1-KO construct (“koESM1”) or the CRISPR/Cas9 control empty vector (“Cas9-C”). Tumor volumes (**A**) and mice body weights (**B**) were recorded every seven days. After 35 days, all tumors were separated (**C**), tumor weights (**D**) and volumes (**E**) were recorded. qRT-PCR (**F**) was used to test the mRNA expression level of *ESM1*, *PI3K*, *Akt*, *E-cadherin*, *N-cadherin*, *Slug*, and *Vimentin*. ESM1 and phosphorylation levels of mTOR, Akt, S6, and 4EBP1 were detected by Western blotting (**G)**. EMT proteins were tsted and results were quantified (**H**). Immunohistochemistry (IHC) staining was used to test the expression of ESM1, Ki67, and p-Akt (Ser-473) in xenograft slides (**I**). The tissue slide TUNEL staining assays were conducted to test cell apoptosis in koESM1 xenografts and Cas9-C xenografts (**J**). The data were presented as mean ± standard deviation (SD). **P < 0.01, ***P < 0.001, ****P < 0.0001 vs. “Cas9-C” xenografts. Scale bar = 25/100 μm.

The mice xenograft model was applied to study the potential effect of ESM1 on cervical cancer cell growth in vivo. At 6 × 10^6^ cells per mouse, SiHa cells bearing the lentiviral CRISPR/Cas9-ESM1-KO construct (“ko-ESM1”) or Cas9-C control vector (“Cas9-C”) were subcutaneously (*s.c*.) injected to the flanks of the nude mice to establish xenograft tumors. The ko-ESM1 SiHa xenografts grew significantly slower than the Cas9-C xenografts (Fig. 6A). There was no significant difference in the animal body weights (Fig. 6B). Thirty-five days after subcutaneous injection, tumors were all removed (Fig. 6C). ko-ESM1 SiHa xenografts were significantly lighter (Fig. 6D) and smaller (Fig. 6E) than Cas9-C SiHa xenografts.

Next, three tumor xenografts of each group were separated carefully and fresh tumor tissue lysates were tested. *ESM1* mRNA and protein levels were significantly reduced in ko-EMS1 xenografts (Fig. 6F and G). In addition, phosphorylation of mTOR, Akt, S6, and 4EBP1, as well as expression of N-Cadherin, Vimentin, and Slug were decreased in ko-EMS1 xenografts tissues(Fig. 6G and H). Whereas E-Cadherin protein expression was increased (Fig. 6H). Furthermore, immunohistochemistry (IHC) staining assay in xenograft slides found that ESM1, Ki67, and p-Akt (Ser-473) were significantly downregulated in ko-ESM1 xenografts (Fig. 6I), where TUNEL-positive nuclei were increased (Fig. 6J). Thus, these results implied that ESM1 KO inhibited PI3K-Akt-mTOR activation, downregulated EMT-promoting proteins, and activated apoptosis in cervical cancer xenografts in vivo.

## Discussion

Accumulating studies have implied the correlation of ESM1 with carcinogenesis and tumor progression [21, 28, 37]. Liu et al., demonstrated that overexpression of ESM1 was closely associated with vascular invasion and distant metastasis in gastric cancer, showing a possible independent poor prognostic factor of ESM1 overexpression in gastric cancer [38]. ESM1 was identified as a potential serum marker for the early detection of colorectal cancer, participating in cell survival, invasion, and EMT through Akt-dependent inhibition of NF-κB/IκB pathway [27]. Liu *et al*., have shown that ESM1 was overexpressed in triple-negative breast cancer, promoting cell proliferation, migration, and invasion by activating Akt/NF-κB/CyclinD1 axis [28]. A very recent study reported that ESM1 overexpression was associated with poor overall survival in metastatic prostate cancer [19]. Pan et al. further showed that ESM1 could accumulate in the nucleus and was associated with prostate cancer stemness [19]. A long noncoding RNA HULC (highly upregulated in liver cancer) was positively correlated with ESM1 in human gliomas. HULC silencing suppressed glioma cell proliferation and invasion, which were reversed after ESM1 overexpression [29].

ESM1 could be a novel and essential oncotarget protein for cervical cancer. First, the number of ESM1 transcripts is significantly higher in TCGA-cervical cancer database, correlating with poor survival, tumor-stage, tumor-grade, and lymphatic vascular invasion. Moreover, *ESM1* mRNA and protein expression are upregulated in local cervical cancer tissues and various human cervical cancer cells. ESM1 shRNA or KO robustly inhibited cervical cancer cell viability, proliferation, migration, invasion as well as cell cycle progression, while inducing apoptosis. Conversely, ectopic overexpression of ESM1 did opposite functions and exerted cancer-promoting activity in cervical cancer cells. In vivo, the growth of SiHa xenografts was largely suppressed after ESM1 knockout.

Cell-cell junctions enable different types of cell movements, coordinating individual-cell migration and streaming, critical for cell proliferation and migration [39]. Defects in cell-cell junctions, commonly detected in various cancers, alter cell stability [40]. EMT enables cancer cell invasion and metastasis through weakening or fully dissolving cell-cell junctions [41–43]. ESM1 is an important player in EMT during tumor progression [27]. Gu *et al*. have shown the expression of EMT-associated proteins could be regulated by ESM1/HIF-1α pathway [26]. ESM1 could also exert a pro-angiogenesis effect via PI3K/Akt/mTOR signaling in human gliomas [29].

Our present study implied ESM1-driven cervical cancer cell progression and EMT by mediating SYT13-dependent activation of PI3K/Akt cascade. In cervical cancer cells, PI3K/Akt activation was largely inhibited by ESM1 shRNA or KO but was augmented following ESM1 overexpression. In vivo, PI3K-Akt activation was also decreased in ko-ESM1 SiHa xenografts. Moreover, ESM1 depletion upregulated E-Cadherin, but downregulated N-Cadherin, vimentin, and Slug in human cervical cancer cells and SiHa xenografts tissues. Ectopic overexpression of ESM1 did the opposite functions.

To further investigate how ESM1 modulated EMT and PI3K-Akt signaling pathways, bioinformatics analysis was performed in RNA-sequencing data to search for the key downstream protein of ESM1. SYT13 is a promising DEG that could be key downstream of ESM1.

Located on human chromosome 11p11.2, *SYT13* encodes a single-pass 47-kDa transmembrane protein [44]. Studies have reported the potential oncogenic role of SYT13 in human cancer [45, 46]. SYT13 knockdown decreased proliferation and metastasis of lung adenocarcinoma cells and gastric cancer cells [47, 48]. Nakanishi *et al*. have shown that SYT13 expression is significantly increased in peritoneal lavage of gastric cancer patients, correlating with poor peritoneal recurrence-free survival and overall survival [48]. Kanda *et al*., showed that intra-abdominal administration of anti-SYT13 antisense oligonucleotides (ASO) dramatically reduced gastric cancer metastasis in a mouse model, and significantly increased animal survival [49].

SYT13 could play an important role in regulating multiple signal cascades in human cancer. Ichikawa et al., reported that SYT13 overexpression in estrogen receptor (ER)-positive breast cancer correlated with several key oncogenes, including estrogen receptor 1 (ER1), Akt, and cyclin-dependent kinases 4 (CDK4) [46]. Kanda *et al*., found SYT13 silencing inhibited activation of multiple pro-cancerous signalings, including cyclin-dependent kinase 2 (CDK2), focal adhesion kinase (FAK) and more importantly PI3K-Akt cascade, without affecting JAK-STAT and NF-κB signalings [49].

Here, the bioinformatics studies and RNA sequencing data revealed that SYT13 could be a primary target of ESM1 in cervical cancer. SYT13 is upregulated in cervical cancer patients, and its overexpression correlated with poor overall survival. Ectopic ESM1 overexpression in cervical cancer cells increased SYT13 expression. Whereas siRNA-induced silencing of SYT13 decreased PI3K-Akt cascade in cervical cancer cells, without affecting ESM1 expression. Importantly, SYT13 silencing potently inhibited ESM1-overexpression-induced PI3K-Akt activation and cervical cancer cell migration/invasion. Therefore, ESM1-driven cervical cancer progression could be due to promoting SYT13 expression. The underlying mechanisms may warrant further characterizations.

Cancer screening, HPV vaccine application combining with the current treatment strategies, including neoadjuvant chemotherapy and immunotherapy, have made cervical cancer a potential preventable and treatable disease (mainly in developed countries) [3, 50]. Yet, for low-middle-income countries, it is still a deadly disease for many patients [4, 5]. Indeed, eighty-five percent of cervical cancer deaths occur in the developing countries [4, 5]. The prognosis and overall survival of the advanced or metastatic cervical cancer are still not satisfactory [4, 5]. According to the global cancer statistics, its incidence ranks 14th among all malignancy and 4th among female cancers [4, 5], and it is the second most common cancer among women in developing countries [4, 5]. It is therefore extremely important to further explore the underlying mechanisms and key signaling proteins for cervical cancer progression [10, 11, 51]. The results of the present study support that overexpressed ESM1 is important for cervical cancer progression, possibly by promoting PI3K-Akt activation and EMT progression. ESM1 therefore represents as a promising diagnostic marker and potential therapeutic target of cervical cancer. Future studies will be needed to possibly develop small-molecule inhibitors or antibodies against ESM1, and to test their efficiency against cervical cancer.

## Materials and Methods

### Chemicals and reagents

Cell Counting Kit-8 (CCK-8) was purchased from Dojindo Co. (Kumamoto, Japan). Puromycin was provided by Sigma-Aldrich Chemicals (St. Louis, Mo). Antibodies for anti-cleaved caspase-3 (#9664), anti-cleaved-poly (ADP-ribose) polymerase (PARP) (#5625), anti-cleaved-caspase-9 (#20750), anti-E-Cadherin (#3195), anti-p-Akt S473 (#4060) and antiphospho-S6 (#4858) were provided by Cell Signaling Tech (Danvers, MA). Anti-ESM1(#103590), anti-N-Cadherin (#245117), anti-Vimentin (#92547), anti-slug (#302780) and all other antibodies were obtained from Abcam (Cambridge, MA). RNA reagents and other transfection reagents were obtained from Thermo-Fisher Invitrogen (Carlsbad, CA). Annexin V and propidium iodide (PI) were purchased from BD Biosciences (Franklin Lakes, NJ).

### Bioinformatics analysis

The Cancer Genome Atlas (TCGA) database (https://gdc.xenahubs.net) was downloaded to analyze ESM1 transcript expression in cervical cancer tissues and normal cervical tissues, and GSE9750 dataset was used to further verify the results. Additionally, Kaplan-Meier survival analysis was used to determine the prognosis of cervical cancer patients. Subgroup analysis by different clinical characterizations was performed based on TCGA database. Gene Ontology (GO) and Kyoto Encyclopedia of Genes and Genomes (KEGG) analysis were conducted by Gene Set Enrichment Analysis (GSEA).

### Cell culture

Two established human cervical cancer cell lines, SiHa and HeLa, and normal cervical epithelial cells (HcerEpic) were purchased from the Cell Bank of Shanghai Institute of Biological Science (Shanghai, China). The primary human cervical cancer cells (priCC-1), derived from one written-informed consent patient at age 55 in authors’ institutions with gynecology and obstetrics (FIGO) stages IIB cervical cancers (squamous cell carcinoma), were cultured as described [52]. Cells were cultivated in DMEM medium (Gibco, Waltham, MA, USA) with 10% fetal bovine serum (FBS, Gibco, Waltham, MA) and 1% penicillin/streptomycin (Gibco, Waltham, MA). The medium was refreshed every three days. The protocols of using primary human cells were approved by Ethics committee of Affiliated Kunshan Hospital of Jiangsu University.

### Human cervical cancer tissues

The cervical cancer tissues and surrounding normal cervical tissues were obtained from 12 primary cervical cancer patients with written-informed consent (administrated at Affiliated Kunshan Hospital of Jiangsu University). Tissues were homogenized by the tissue lysis buffer (Beyotime Biotechnology, Wuxi, China), followed by further biochemical analyses. The protocols of this study comply with the Declaration of Helsinki and were approved by the Ethics committee of Affiliated Kunshan Hospital of Jiangsu University.

### ESM1 shRNA

Three lentiviral shRNAs targeting non-overlapping sequences of human ESM1(“shESM1-a”, “shESM1-b”, and “shESM1-c”), as well as the scramble control (“shC”) were designed and validated by Genechem Co. Cells were inoculated into six-well plates in complete medium, and transfected with ESM1 shRNAs and scramble control respectively. Stable cells were screened for two weeks in complete medium with puromycin (5.0 µg/mL). Silencing of ESM1 in stable cells was verified by qPCR and Western blotting.

### CRISPR/Cas9 knockout of ESM1

Lentiviral CRISPR/Cas-9 ESM1 KO construct and CRISPR/Cas-9 empty vector, provided by Genechem, were transfected into SiHa and HeLa cells [53]. ESM1 KO stable cells were screened with puromycin and verified by qPCR and Western blotting.

### Forced ESM1 overexpression

The full-length ESM1 cDNA-expressing lentiviral construct and the empty vector, provided by Genechem (Shanghai, China), were transfected into SiHa and HeLa cells. After being selected by puromycin, ESM1 overexpression in stable cells was verified by qPCR and Western blotting.

### RNA interference (RNAi)

ESM1 overexpressed cervical cancer cells were transfected with a small interference RNA (siRNA) targeting SYT13 and negative control siRNA by Lipofectamine 2000 (Invitrogen, Carlsbad, CA). 48 h after transfection, cells were used for further experiments.

### Xenograft assay

Severe combined immunodeficiency (SCID) mice (15-16 g, female) were purchased from the Animal Center of Jiangsu University (Zhenjiang, China). These mice were assigned into two random groups. Genetically modified SiHa cells were resuspended in Matrigel-containing serum-free medium, then injected subcutaneously into the right flanks of nude mice. Body weights and tumor volumes were recorded every seven days. Immunohistochemistry (IHC) staining was conducted by the procedure described previously [54]. Animal experiments were approved by the Institutional Animal Care and Use Committee (IACUC) and Ethics Committee of Affiliated Kunshan Hospital of Jiangsu University.

### Cellular functional studies

Cell counting Kit-8 (CCK-8) cell viability assay, colony formation assay, nuclear 5-ethynyl-2’-deoxyuridine (EdU) staining assay, wound healing assay, “Transwell” and “Matrigel Transwell” analyses were performed following the protocols described previously [55–59]. Propidium iodide staining and fluorescence-activated cell sorting (PI-FACS) assaying of cell cycle progression and apoptosis, Western blotting analysis, as well as RNA extraction and quantitative reverse transcription-PCR (qRT-PCR) were also described previously [55–57]. The uncropped blotting images were shown in Figure S1.

### Immunohistochemistry (IHC) staining and Terminal deoxynucleotidyl transferase (TdT) dUTP Nick-End Labeling (TUNEL) assay

The IHC staining protocol has been reported in Liu et al. [54]. TUNEL staining was performed on 4% paraformaldehyde-fixed, paraffin-embedded xenograft tissue sections. Slices were stained with terminal deoxynucleotidyl transferase dUTP Labeling buffer and DAPI following manufacture instructions. Finally, slices were sealed and photographed under an OlympusFSX100 microscope (Olympus, Tokyo, Japan).

### Statistical analyses

All statistical analyses were performed using SPSS 22.0 (IBM) and R (R version3.5.1; Institute for Statistics and Mathematics, Vienna, Austria). All data were with normal distribution and were presented as means ± standard deviation (SD) from at least three separate experiments. The differences among the groups of each treatment were analyzed by one-way ANOVA (three or more groups) or Student’st-test (two groups). RNA sequencing data was analyzed using R. software (version 4.2.1). “limma” package, “clusterprofiler” package, “ggplot2” package, “survival” package, and “survminer” package were installed and used. The difference was considered statistically significant as *P* value <0.05.

## Conclusion

In conclusion, overexpressed ESM1 is important for cervical cancer growth in vitro and in vivo, possibly by promoting PI3K-Akt activation and EMT progression.

## Supplementary information

Supplementary Figures

aj-checklist form

Author contribution form

## Data Availability

All data are available upon request.
