# Supplementary material for: Endothelial cell-specific molecule 1 drives cervical cancer progression
Source: Cell Death Dis. 2022 Dec 15;13(12):1043. doi: 10.1038/s41419-022-05501-5 (PMC9755307; doi:10.1038/s41419-022-05501-5)
Supplement: Supplementary file 1 — Supplementary Figures [file 41419_2022_5501_MOESM1_ESM.pdf]

Figure S1: The un-cropped blotting images of the present study.

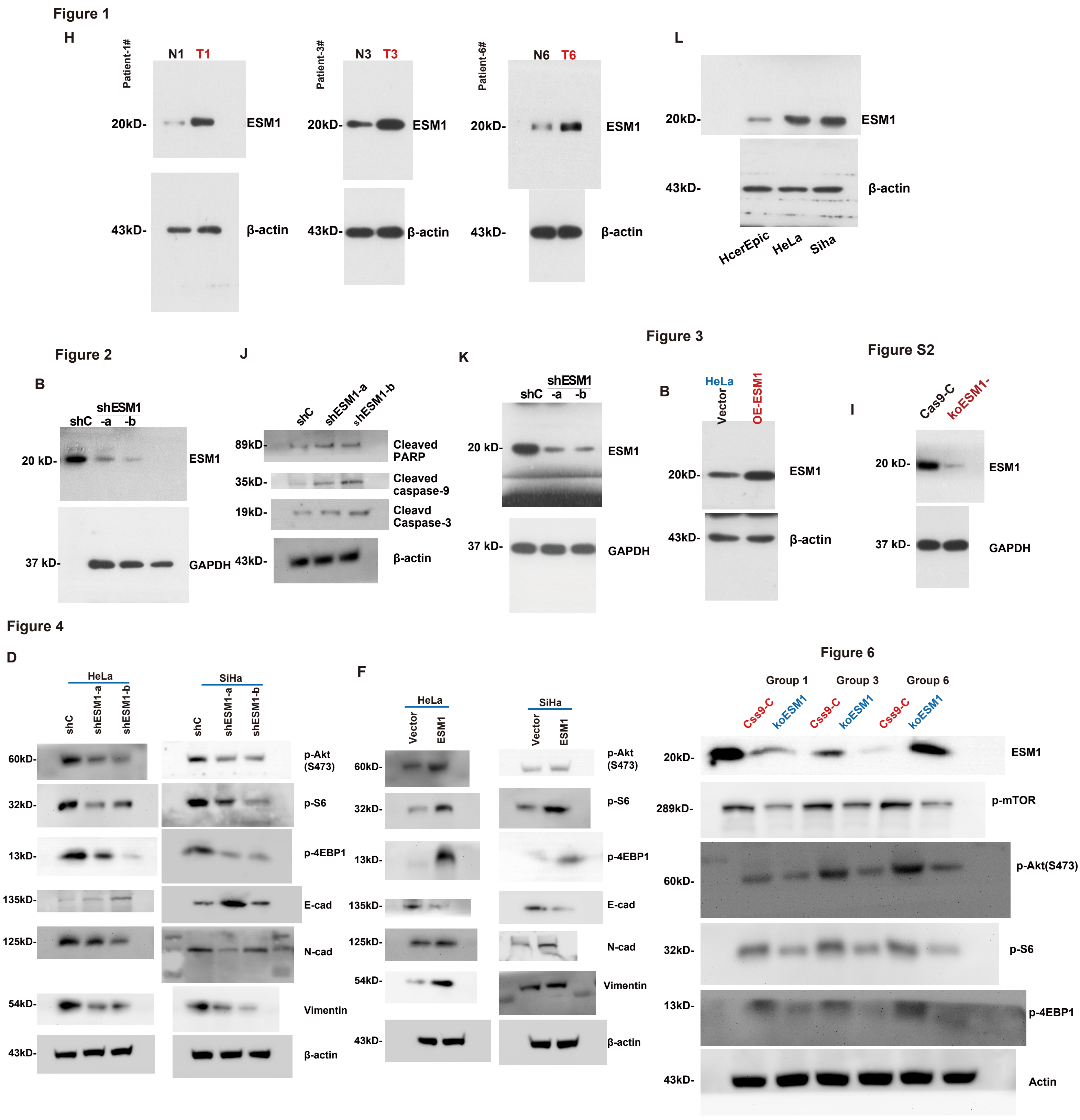

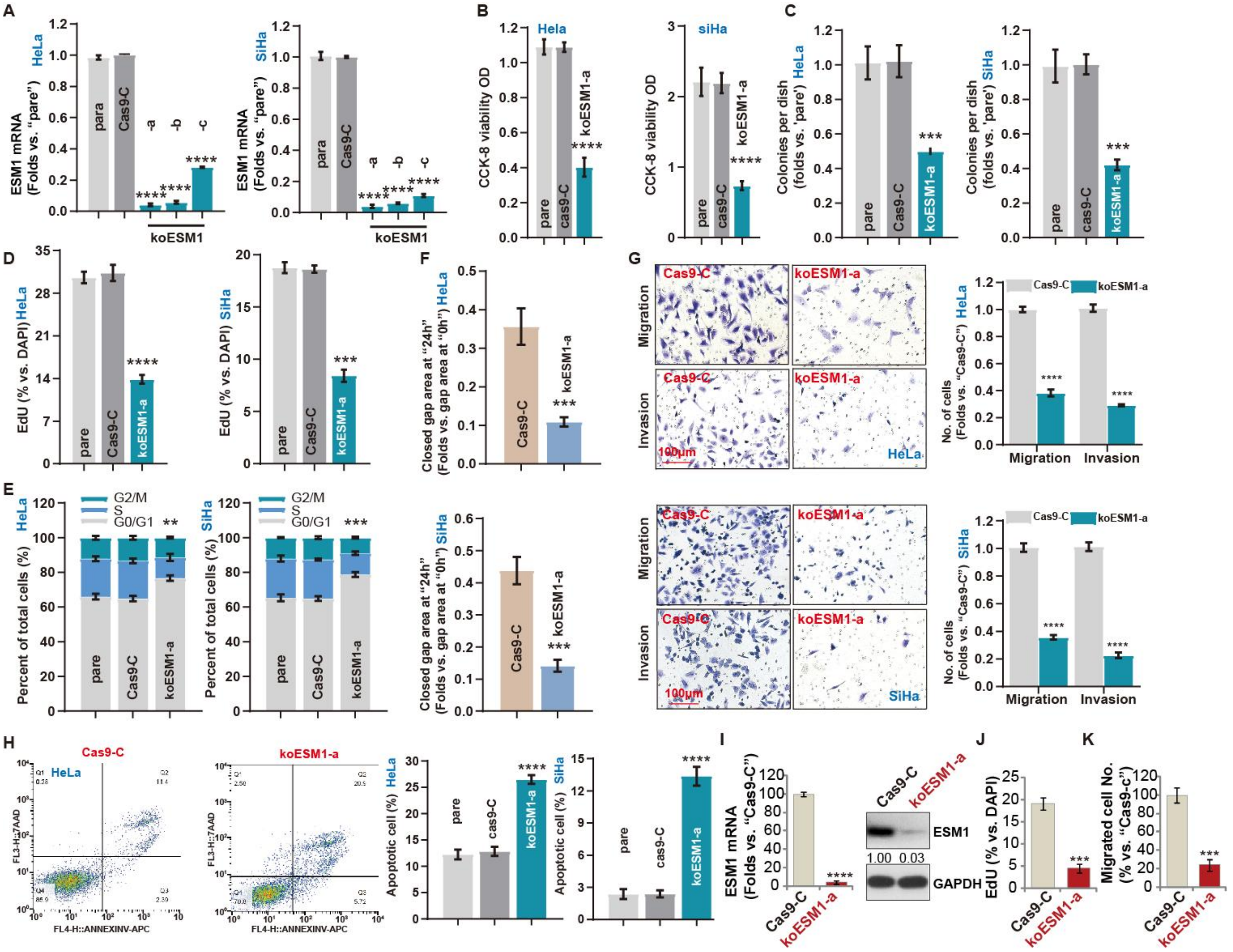

Fig S2. CRISPR/Cas9-mediated ESM1 knockout inhibits the progression of cervical cancer cells. Established cervical cancer cell lines (HeLa and SiHa) (**A-H**) and primary cervical cancer cells ("priCC-1") (**I-K**) bearing the lentiviral CRISPR/Cas9-ESM1-KO construct ("koESM1") or the CRISPR/Cas9 control empty vector ("Cas9-C"), as well as the parental control cells ("pare"), were established and cultured for applied time periods. ESM1 KO was confirmed by qRT-PCR (**A** and **I**) and Western blotting (**I**) assays. Cell viability (**B**), colony formation (**C**), proliferation (**D** and **J**), cell cycles progression (**E**), cell migration and cell invasion (**F**, **G** and **K**), as well as cell apoptosis (**H**) were tested by the listed assays, with results quantified. The data were presented as mean ± standard deviation (SD, n = 3). * P < 0.05, **P <0.01, *** P < 0.001, **** P < 0,0001 vs. "Cas9-C" cells. The experiments were repeated three times with similar results obtained. Scale bar = 100 μm.
