# Supplementary material for: Endothelial cell-specific molecule 1 drives cervical cancer progression
Source: Cell Death Dis. 2022 Dec 15;13(12):1043. doi: 10.1038/s41419-022-05501-5 (PMC9755307; doi:10.1038/s41419-022-05501-5)
Supplement: Supplementary file 3 — Author contribution form [file 41419_2022_5501_MOESM3_ESM.pdf]

DECLARATION OF CONTRIBUTIONS TO ARTICLE

ADMC

Manuscript Number:	Journal Name:	
CDDIS-22-3030RR	*Cell Death & Disease*	(the 'Journal')

Proposed Title of the Contribution:

Endothelial cell-specific molecule 1 drives cervical cancer progression (the 'Contribution)

Author(s):

Jingjing Lu, Qin Liu, Lixia Zhu, Yuanyuan Liu, Xiaoren Zhu, Shiqing Peng, Minbin Chen and Ping Li (the 'Authors')

For all *CDDis* articles, each person named as an author in the published version must be able to show he or she has contributed substantially to the article.

Authorship credit should be based on 1) substantial contributions to conception and design, acquisition of data, or analysis and interpretation of data; 2) drafting the article or revising it critically for important intellectual content; and 3) final approval of the version to be published. Authors should meet conditions 1, 2 and 3.

Any person who cannot be shown to have made a substantial contribution to the article cannot be listed as an author in the final version. The name of any person who is deemed to have made a minor contribution can, however, appear in the Acknowledgments section of the article.

Please complete the table below to indicate the contributions of all named authors to the manuscript.

Author Full Name:	Specification of Contribution to the Manuscript:
Jingjing Lu	Functional assays in cancer and normal cells, establishing xenografts in mice, testing signalings in cells and tissues and performing cell death/apoptosis assays, drafting the article and revising it, with final approval of the version submitted to the journal.
Qin Liu	Functional assays in cancer and normal cells, establishing xenografts in mice, testing signalings in cells and tissues and performing cell death/apoptosis assays, drafting the article and revising it, with final approval of the version submitted to the journal.
Lixia Zhu	Preparing and verifying the virus, sequences, constructs, and primers, drafting the article and revising it, with final approval of the version submitted to the journal.
Yuanyuan Liu	Performing cell death/apoptosis assays, drafting the article and revising it, with final approval of the version submitted to the journal.
Xiaoren Zhu	Bioinformatics studies, drafting the article and revising it, with final approval of the version submitted to the journal.
Shiqing Peng	Bioinformatics studies, drafting the article and revising it, with final approval of the version submitted to the journal.
Minbin Chen	Study conception and design, and data analysis, Figure organization, involved in drafting the article and revising it critically for important intellectual content, and with final approval of the version submitted to the journal.
Ping Li	Study conception and design, and data analysis, Figure organization, involved in drafting the article and revising it critically for important intellectual content, and with final approval of the version submitted to the journal.

Please complete the table below to indicate the contributions of all named authors to the figures.

Figure 1:

Jingjing Lu, Qin Liu, Lixia Zhu, Yuanyuan Liu, Xiaoren Zhu, Shiqing Peng, Minbin Chen and Ping Li

Figure 2:

Jingjing Lu, Qin Liu, Lixia Zhu, Yuanyuan Liu, Xiaoren Zhu, Shiqing Peng, Minbin Chen and Ping Li

Figure 3:

Jingjing Lu, Qin Liu, Lixia Zhu, Yuanyuan Liu, Xiaoren Zhu, Shiqing Peng, Minbin Chen and Ping Li

Figure 4:

Jingjing Lu, Qin Liu, Lixia Zhu, Yuanyuan Liu, Xiaoren Zhu, Shiqing Peng, Minbin Chen and Ping Li

Figure 5:

Jingjing Lu, Qin Liu, Lixia Zhu, Yuanyuan Liu, Xiaoren Zhu, Shiqing Peng, Minbin Chen and Ping Li

Figure 6:

Jingjing Lu, Qin Liu, Lixia Zhu, Yuanyuan Liu, Xiaoren Zhu, Shiqing Peng, Minbin Chen and Ping Li

Signed for and on behalf of the Author(s):	Print Name:	Date:
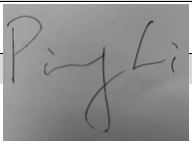	Ping Li	12-06-2022
